# Ethyl 4-{1-[(2,4-dinitro­phen­yl)hydrazono]eth­yl}-5-(2-naphthyl­methoxy­meth­yl)isoxazole-3-carboxyl­ate

**DOI:** 10.1107/S1600536808041901

**Published:** 2008-12-17

**Authors:** Nicholas R. Natale, Monica I. Szabon-Watola, Brendan Twamley, Richard J. Bridges, Sarjubhai Patel, Trideep Rajale

**Affiliations:** a Center For Structural and Functional Neuroscience, Department of Biomedical & Pharmaceutical Sciences, University of Montana, Missoula, MT 59812, USA; b Department of Chemistry, University of Idaho, Moscow, ID 83844-2343, USA

## Abstract

The title compound, C_26_H_23_N_5_O_8_, was prepared and its structure investigated to further develop a working hypothesis for the essential binding pharmacophore for ligands of the System Xc- transporter [Patel *et al.* (2004 ▶). *Neuropharmacology*, **46**, 273–284]. The hydrazone group displays an *E* geometry and the isoxazole double bond and C=N group of the hydrazone are in an *s*-*cis* relationship. The secondary amino NH group forms an intra­molecular N—H⋯O hydrogen bond to a ring nitro group. There is a dihedral angle of 44.27 (5)° between the isoxazole plane and the hydrazone group plane.

## Related literature

For a related structure, see: Burkhart *et al.* (1999 ▶, 2001 ▶). For general background, see: Davis *et al.* (1993 ▶); Honore & Lauridsen (1980 ▶); Krogsgaard-Larsen, Honore, Hansen, Curtis & Lodge (1980 ▶); Natale *et al.* (2006 ▶); Patel *et al.* (2004 ▶, 2006 ▶); Stables & Kupferberg (2008 ▶); Twamley *et al.* (2007 ▶); Zhou & Natale (1998 ▶).
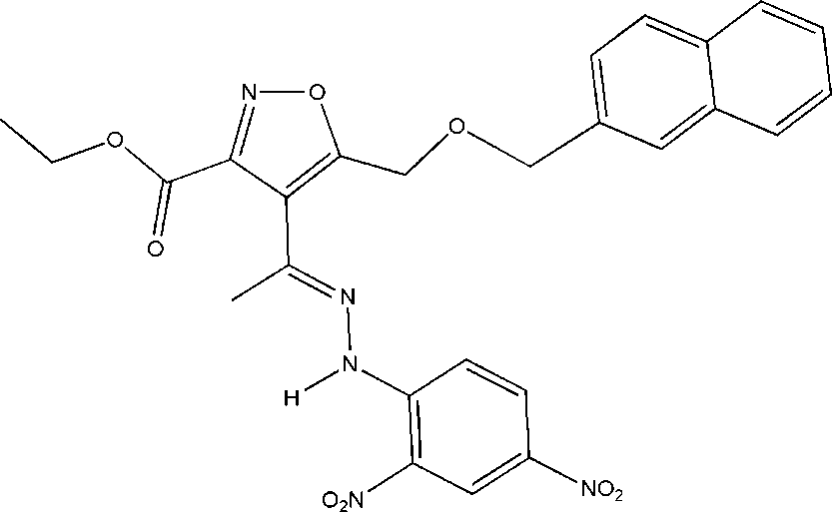


## Experimental

### 

#### Crystal data

C_26_H_23_N_5_O_8_
*M* *_r_* = 533.49
Triclinic, 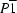

*a* = 7.0839 (6) Å
*b* = 12.176 (1) Å
*c* = 14.184 (2) Å
α = 90.581 (1)°
β = 95.925 (2)°
γ = 99.251 (2)°
*V* = 1200.7 (2) Å^3^
*Z* = 2
Mo *K*α radiation
μ = 0.11 mm^−1^
*T* = 90 (2) K
0.47 × 0.33 × 0.30 mm

#### Data collection

Bruker SMART APEX diffractometer
Absorption correction: multi-scan (*SADABS*; Bruker, 2007 ▶) *T*_min_ = 0.949, *T*_max_ = 0.971
18560 measured reflections
4357 independent reflections
4009 reflections with *I* > 2σ(*I*)
*R*_int_ = 0.023

#### Refinement

*R*[*F* ^2^ > 2σ(*F* ^2^)] = 0.035
*wR*(*F* ^2^) = 0.092
*S* = 1.02
4357 reflections
354 parameters
H-atom parameters constrained
Δρ_max_ = 0.28 e Å^−3^
Δρ_min_ = −0.23 e Å^−3^

### 

Data collection: *SMART* (Bruker, 2007 ▶); cell refinement: *SAINT-Plus* (Bruker, 2007 ▶); data reduction: *SAINT-Plus*; program(s) used to solve structure: *XS* in *SHELXTL* (Sheldrick, 2008 ▶); program(s) used to refine structure: *XL* in *SHELXTL*; molecular graphics: *XP* in *SHELXTL*; software used to prepare material for publication: *publCIF* (Westrip, 2009 ▶).

## Supplementary Material

Crystal structure: contains datablocks global, I. DOI: 10.1107/S1600536808041901/hg2449sup1.cif

Structure factors: contains datablocks I. DOI: 10.1107/S1600536808041901/hg2449Isup2.hkl

Additional supplementary materials:  crystallographic information; 3D view; checkCIF report

## Figures and Tables

**Table 1:** Hydrogen-bond geometry (Å, °)

*D*—H⋯*A*	*D*—H	H⋯*A*	*D*⋯*A*	*D*—H⋯*A*
N28—H28*A*⋯O37	0.88	1.96	2.6028 (14)	128

**Fig. 1.**
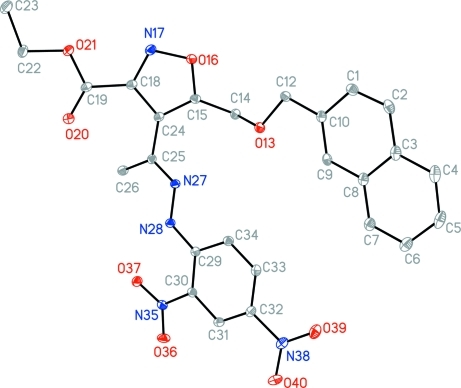
Molecular Structure of (Ia), showing 30% probablility displacement ellipsoids.

**Fig. 2.**
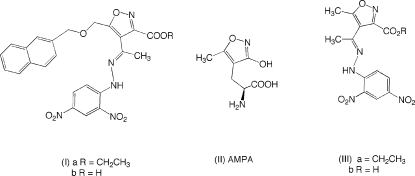
Structure of the title Compound (Ia), corresponding carboxylic acid (Ib), the neurotransmitter AMPA (II), and previously reported simple analog (III).

## supplementary crystallographic information

### Comment

In the course of our continuing studies on the synthesis and structure activity relationships of analogs of AMPA (II) (see Figure 2; Krogsgaard-Larsen *et al.*, 1980; Honore, & Lauridsen, 1980) for glutamate receptors and transporters (Natale *et al.*, 2006), we have found that a simple isoxazole hydrazone (IIIb) (Burkhart *et al.*, 1999) exhibited significant binding at the System Xc- transporter (SXc-) (Patel *et al.*, 2004), and that this correlated with anticonvulsant activity *in vivo* (Stables & Kupferberg, 2008). Since the three dimensional structure of the SXc- is unsolved at this writing we have developed a preliminary pharmacophore model for ligand binding which indicates that lipophilic groups appear to be tolerated (Patel *et al.*, 2006), which is also promising from the perspective of increasing the likelihood of delivering such ligands past the blood brain barrier. Therefore we carried out the synthesis of (Ia) and also examined its structure, see Figure 1. We had previously examined the structure of (IIIa), (see Figure 2) and found it adopted an s-*trans*-E geometry at the juncture between the isoxazole and the hydrazone double bond, respectively (Burkhart *et al.*, 1999). The naphthyloxy analog (Ia) adopts a similar E-geometry at the C=N double bond, but an s-*cis* conformation at the C-4 bond between the isoxazole and the hydrazone. The observation that (Ib) exhibits no significant glutamate inhibition at SystemXc- represents a negative control in the Structure Activity Relationship. This raises interesting questions as to the relationship between conformation and geometry vis-a-vis biological effect, and this will be the subject of forthcoming manuscripts.

### Experimental

The title compound (Ia) was prepared from ethyl 5-methyl-4-(2,5,5-trimethyl-1,3-dioxan-2-yl)isoxazole-3-carboxylate (Zhou & Natale, 1998) *via* lateral metalation (Burkhart *et al.*, 2001), and electrophilic quenching using the Davis oxaziridine (Davis *et al.*, 1993), to the corresponding 5-methyl alcohol. This alcohol can also be prepared by bromination followed by nucleophilic substitution by water (Twamley *et al.*, 2007). The title compound was obtained from the 5-methyl alcohol by Williamson ether synthesis, deprotection and hydrazone formation (Burkhart *et al.*, 1999).

4-{1-[(2,4-Dinitro-phenyl)-hydrazono]-ethyl}-5-(naphthalen-2-ylmethoxymethyl)-\ isoxazole-3-carboxylic acid ethyl ester (Ia)

To a stirred solution of ethyl 5-(naphthalen-2-yl-methoxymethyl)-4-acetyl-isoxazole-3-carboxylate (0.650 g, 1.93 mmol), in 10 ml of THF, a solution of 12 ml (1.0 eq.) of reagent 2,4-DNP was added and the reaction mixture was monitored by TLC (ether/hexane as a mobile phase). During reaction the reddish precipitate formed which was separated and purified by column chromatography. The fast moving, major isomer was examined by crystallography. Yield 57% The major isomer, yellow crystals, m.p.= 105–107 °C, ^1^H NMR (deuteriochloroform): δ 1.45 (t, 3H, J=7.1 Hz), 2.34 (s, 3H), 4.48 (q, 2H, J=7.1 Hz), 4.77 (s, 2H), 4.83 (s, 2H), 7.42 (m, 3H), 7.56 (d, 1H, J=9.5 Hz), 7.80 (m, 4H), 8.03 (dd, 1H, J=2.4, 9.5 Hz), 9.00 (d, 1H, J=2.6 Hz), 9.99 (brs, 1H, NH). ^13^C NMR (500 MHz) δ 14.1, 17.3, 60.6, 62.7, 73.1, 116.0, 118.3, 123.2, 125.4, 126.3, 126.4, 127.0, 127.5, 127.6, 128.4, 129.8, 132.9, 133.0, 134.1, 138.6, 143.8, 144.2, 154.1, 159.8, 168.8. The minor isomer, ^1^H NMR (deuteriochloroform):δ 1.40 (t, 3H, J=7.1 Hz), 2.43 (s, 3H), 4.46 (q, 2H, J=7.1 Hz), 4.80 (s, 2H), 4.93 (s, 2H), 7.22 (d, 1H, J=9.5 Hz), 7.42 (m, 3H), 7.80 (m, 4H), 8.03 (dd, 1H, J=2.4, 9.5 Hz), 8.65 (d, 1H, J=2.6 Hz), 10.75 (brs, 1H, NH).

### Refinement

All other H atoms were positioned geometrically and refined using a riding model, with U_iso_ constrained to be 1.2U_eq_ (CH_arom_, CH_2_ = 0.95–0.99 Å) and 1.5U_eq_ (CH_3_ = 0.98Å) of the carrier atom.

### Crystal data

**Table:**

C_26_H_23_N_5_O_8_	*Z* = 2
*M**_r_* = 533.49	*F*(000) = 556
Triclinic, *P*1	*D*_x_ = 1.476 Mg m^−^^3^
Hall symbol: -P 1	Mo *K*α radiation, λ = 0.71073 Å
*a* = 7.0839 (6) Å	Cell parameters from 6135 reflections
*b* = 12.176 (1) Å	θ = 2.3–30.1°
*c* = 14.184 (2) Å	µ = 0.11 mm^−^^1^
α = 90.581 (1)°	*T* = 90 K
β = 95.925 (2)°	Needle, yellow
γ = 99.251 (2)°	0.47 × 0.33 × 0.30 mm
*V* = 1200.7 (2) Å^3^	

### Data collection

**Table:**

Bruker SMART APEX diffractometer	4357 independent reflections
Radiation source: normal-focus sealed tube	4009 reflections with *I* > 2σ(*I*)
graphite	*R*_int_ = 0.023
Detector resolution: 8.3 pixels mm^-1^	θ_max_ = 25.3°, θ_min_ = 2.2°
ω scans	*h* = −8→8
Absorption correction: multi-scan (*SADABS*; Bruker, 2007)	*k* = −14→14
*T*_min_ = 0.949, *T*_max_ = 0.971	*l* = −17→17
18560 measured reflections	

### Refinement

**Table:**

Refinement on *F*^2^	Primary atom site location: structure-invariant direct methods
Least-squares matrix: full	Secondary atom site location: difference Fourier map
*R*[*F*^2^ > 2σ(*F*^2^)] = 0.035	Hydrogen site location: inferred from neighbouring sites
*wR*(*F*^2^) = 0.092	H-atom parameters constrained
*S* = 1.02	*w* = 1/[σ^2^(*F*_o_^2^) + (0.0462*P*)^2^ + 0.5022*P*] where *P* = (*F*_o_^2^ + 2*F*_c_^2^)/3
4357 reflections	(Δ/σ)_max_ < 0.001
354 parameters	Δρ_max_ = 0.28 e Å^−^^3^
0 restraints	Δρ_min_ = −0.23 e Å^−^^3^

### Special details

**Table:**

Geometry. All e.s.d.'s (except the e.s.d. in the dihedral angle between two l.s. planes) are estimated using the full covariance matrix. The cell e.s.d.'s are taken into account individually in the estimation of e.s.d.'s in distances, angles and torsion angles; correlations between e.s.d.'s in cell parameters are only used when they are defined by crystal symmetry. An approximate (isotropic) treatment of cell e.s.d.'s is used for estimating e.s.d.'s involving l.s. planes.
Refinement. Refinement of *F*^2^ against ALL reflections. The weighted *R*-factor *wR* and goodness of fit *S* are based on *F*^2^, conventional *R*-factors *R* are based on *F*, with *F* set to zero for negative *F*^2^. The threshold expression of *F*^2^ > σ(*F*^2^) is used only for calculating *R*-factors(gt) *etc*. and is not relevant to the choice of reflections for refinement. *R*-factors based on *F*^2^ are statistically about twice as large as those based on *F*, and *R*- factors based on ALL data will be even larger.

### Fractional atomic coordinates and isotropic or equivalent isotropic displacement parameters (Å^2^)

**Table:**

	*x*	*y*	*z*	*U*_iso_*/*U*_eq_	
C1	0.86385 (19)	0.87869 (12)	0.65591 (10)	0.0225 (3)	
H1A	0.8404	0.9531	0.6498	0.027*	
C2	0.9219 (2)	0.84176 (12)	0.74301 (10)	0.0243 (3)	
H2A	0.9396	0.8910	0.7967	0.029*	
C3	0.95619 (18)	0.73084 (12)	0.75441 (10)	0.0223 (3)	
C4	1.0160 (2)	0.68877 (14)	0.84368 (10)	0.0279 (3)	
H4A	1.0362	0.7363	0.8985	0.034*	
C5	1.0451 (2)	0.58036 (14)	0.85179 (11)	0.0300 (4)	
H5A	1.0835	0.5532	0.9121	0.036*	
C6	1.01822 (19)	0.50944 (13)	0.77116 (11)	0.0272 (3)	
H6A	1.0391	0.4346	0.7772	0.033*	
C7	0.96224 (19)	0.54760 (12)	0.68412 (10)	0.0230 (3)	
H7A	0.9457	0.4990	0.6301	0.028*	
C8	0.92856 (18)	0.65841 (11)	0.67329 (10)	0.0199 (3)	
C9	0.86964 (18)	0.69966 (11)	0.58372 (9)	0.0186 (3)	
H9A	0.8517	0.6517	0.5292	0.022*	
C10	0.83807 (18)	0.80712 (11)	0.57424 (9)	0.0187 (3)	
C12	0.78057 (19)	0.85335 (11)	0.47991 (9)	0.0199 (3)	
H12A	0.6616	0.8857	0.4827	0.024*	
H12B	0.8835	0.9130	0.4636	0.024*	
O13	0.74889 (14)	0.76632 (8)	0.40973 (7)	0.0234 (2)	
C14	0.7541 (2)	0.80387 (11)	0.31558 (9)	0.0210 (3)	
H14A	0.8040	0.7487	0.2772	0.025*	
H14B	0.8446	0.8749	0.3162	0.025*	
C15	0.56224 (19)	0.82104 (11)	0.26945 (9)	0.0183 (3)	
O16	0.50754 (14)	0.92145 (7)	0.28170 (7)	0.0218 (2)	
N17	0.32990 (17)	0.92234 (9)	0.22939 (8)	0.0214 (3)	
C18	0.28435 (19)	0.82377 (11)	0.18738 (9)	0.0178 (3)	
C19	0.09928 (19)	0.79318 (11)	0.12615 (9)	0.0183 (3)	
O20	0.04690 (14)	0.70128 (8)	0.09134 (7)	0.0255 (2)	
O21	0.00088 (13)	0.87728 (8)	0.11528 (7)	0.0221 (2)	
C22	−0.1807 (2)	0.85128 (12)	0.05412 (10)	0.0248 (3)	
H22A	−0.2644	0.7876	0.0792	0.030*	
H22B	−0.1561	0.8310	−0.0107	0.030*	
C23	−0.2763 (2)	0.95228 (13)	0.05216 (11)	0.0303 (3)	
H23A	−0.3983	0.9371	0.0112	0.045*	
H23B	−0.1922	1.0148	0.0274	0.045*	
H23C	−0.3012	0.9712	0.1166	0.045*	
C24	0.42658 (18)	0.75465 (11)	0.21070 (9)	0.0166 (3)	
C25	0.43276 (18)	0.63811 (11)	0.18367 (9)	0.0163 (3)	
C26	0.4029 (2)	0.59917 (11)	0.08182 (9)	0.0211 (3)	
H26A	0.2937	0.5381	0.0727	0.032*	
H26B	0.5190	0.5731	0.0647	0.032*	
H26C	0.3763	0.6609	0.0414	0.032*	
N27	0.47840 (15)	0.57795 (9)	0.25405 (8)	0.0166 (2)	
N28	0.49202 (15)	0.47000 (9)	0.23163 (8)	0.0169 (2)	
H28A	0.4652	0.4444	0.1727	0.020*	
C29	0.54745 (17)	0.40296 (10)	0.30165 (9)	0.0159 (3)	
C30	0.56433 (18)	0.29033 (10)	0.28470 (9)	0.0165 (3)	
C31	0.62357 (18)	0.22374 (11)	0.35731 (10)	0.0179 (3)	
H31A	0.6335	0.1482	0.3449	0.022*	
C32	0.66733 (18)	0.26928 (11)	0.44716 (9)	0.0184 (3)	
C33	0.65367 (18)	0.37992 (11)	0.46714 (9)	0.0186 (3)	
H33A	0.6844	0.4096	0.5301	0.022*	
C34	0.59579 (18)	0.44555 (11)	0.39560 (9)	0.0180 (3)	
H34A	0.5880	0.5211	0.4093	0.022*	
N35	0.52605 (15)	0.23912 (9)	0.19029 (8)	0.0186 (2)	
O36	0.56216 (15)	0.14538 (8)	0.17852 (7)	0.0263 (2)	
O37	0.45844 (14)	0.29207 (8)	0.12397 (7)	0.0222 (2)	
N38	0.73724 (16)	0.20206 (10)	0.52367 (8)	0.0215 (3)	
O39	0.79957 (15)	0.24887 (9)	0.60029 (7)	0.0288 (2)	
O40	0.73174 (15)	0.10215 (8)	0.50788 (8)	0.0292 (2)	

### Atomic displacement parameters (Å^2^)

**Table:**

	*U*^11^	*U*^22^	*U*^33^	*U*^12^	*U*^13^	*U*^23^
C1	0.0195 (7)	0.0215 (7)	0.0262 (7)	0.0015 (5)	0.0036 (6)	−0.0018 (6)
C2	0.0201 (7)	0.0306 (8)	0.0208 (7)	0.0002 (6)	0.0033 (5)	−0.0071 (6)
C3	0.0130 (6)	0.0332 (8)	0.0198 (7)	−0.0001 (6)	0.0033 (5)	0.0021 (6)
C4	0.0183 (7)	0.0457 (9)	0.0186 (7)	0.0014 (6)	0.0022 (5)	0.0002 (6)
C5	0.0185 (7)	0.0494 (10)	0.0221 (7)	0.0044 (6)	0.0019 (6)	0.0141 (7)
C6	0.0163 (7)	0.0330 (8)	0.0320 (8)	0.0026 (6)	0.0029 (6)	0.0116 (6)
C7	0.0156 (6)	0.0273 (7)	0.0248 (7)	0.0000 (5)	0.0016 (5)	0.0037 (6)
C8	0.0116 (6)	0.0263 (7)	0.0208 (7)	−0.0010 (5)	0.0031 (5)	0.0029 (5)
C9	0.0140 (6)	0.0234 (7)	0.0176 (7)	0.0002 (5)	0.0014 (5)	−0.0011 (5)
C10	0.0130 (6)	0.0222 (7)	0.0202 (7)	0.0005 (5)	0.0025 (5)	0.0010 (5)
C12	0.0189 (6)	0.0181 (7)	0.0223 (7)	0.0024 (5)	0.0014 (5)	−0.0008 (5)
O13	0.0318 (5)	0.0187 (5)	0.0175 (5)	0.0021 (4)	−0.0043 (4)	0.0013 (4)
C14	0.0244 (7)	0.0205 (7)	0.0175 (7)	0.0031 (5)	−0.0002 (5)	0.0025 (5)
C15	0.0250 (7)	0.0154 (6)	0.0149 (6)	0.0035 (5)	0.0036 (5)	0.0027 (5)
O16	0.0270 (5)	0.0169 (5)	0.0208 (5)	0.0048 (4)	−0.0026 (4)	−0.0004 (4)
N17	0.0248 (6)	0.0205 (6)	0.0196 (6)	0.0067 (5)	−0.0001 (5)	0.0032 (5)
C18	0.0223 (7)	0.0170 (6)	0.0150 (6)	0.0048 (5)	0.0041 (5)	0.0037 (5)
C19	0.0217 (7)	0.0186 (7)	0.0164 (6)	0.0064 (5)	0.0058 (5)	0.0046 (5)
O20	0.0271 (5)	0.0202 (5)	0.0287 (6)	0.0057 (4)	−0.0028 (4)	−0.0007 (4)
O21	0.0218 (5)	0.0216 (5)	0.0240 (5)	0.0083 (4)	−0.0007 (4)	0.0012 (4)
C22	0.0199 (7)	0.0309 (8)	0.0244 (7)	0.0089 (6)	−0.0012 (6)	−0.0018 (6)
C23	0.0261 (8)	0.0322 (8)	0.0344 (8)	0.0122 (6)	−0.0006 (6)	0.0043 (7)
C24	0.0199 (6)	0.0172 (6)	0.0137 (6)	0.0043 (5)	0.0035 (5)	0.0038 (5)
C25	0.0141 (6)	0.0173 (6)	0.0180 (6)	0.0035 (5)	0.0018 (5)	0.0013 (5)
C26	0.0260 (7)	0.0192 (7)	0.0187 (7)	0.0058 (5)	0.0020 (5)	0.0011 (5)
N27	0.0159 (5)	0.0141 (5)	0.0204 (6)	0.0037 (4)	0.0018 (4)	0.0004 (4)
N28	0.0208 (6)	0.0149 (5)	0.0151 (5)	0.0043 (4)	0.0008 (4)	−0.0001 (4)
C29	0.0118 (6)	0.0169 (6)	0.0192 (6)	0.0019 (5)	0.0035 (5)	0.0019 (5)
C30	0.0139 (6)	0.0165 (6)	0.0188 (7)	0.0008 (5)	0.0031 (5)	0.0003 (5)
C31	0.0145 (6)	0.0158 (6)	0.0243 (7)	0.0027 (5)	0.0052 (5)	0.0027 (5)
C32	0.0138 (6)	0.0218 (7)	0.0203 (7)	0.0033 (5)	0.0037 (5)	0.0065 (5)
C33	0.0164 (6)	0.0225 (7)	0.0170 (7)	0.0034 (5)	0.0022 (5)	0.0003 (5)
C34	0.0177 (6)	0.0165 (6)	0.0204 (7)	0.0036 (5)	0.0032 (5)	−0.0004 (5)
N35	0.0179 (6)	0.0166 (6)	0.0212 (6)	0.0014 (4)	0.0036 (4)	−0.0005 (4)
O36	0.0373 (6)	0.0150 (5)	0.0275 (5)	0.0073 (4)	0.0038 (4)	−0.0032 (4)
O37	0.0275 (5)	0.0214 (5)	0.0176 (5)	0.0058 (4)	−0.0010 (4)	0.0007 (4)
N38	0.0183 (6)	0.0251 (6)	0.0230 (6)	0.0066 (5)	0.0058 (5)	0.0078 (5)
O39	0.0328 (6)	0.0356 (6)	0.0190 (5)	0.0098 (5)	−0.0001 (4)	0.0051 (4)
O40	0.0360 (6)	0.0203 (5)	0.0333 (6)	0.0095 (4)	0.0040 (5)	0.0093 (4)

### Geometric parameters (Å, °)

**Table:**

C1—C2	1.365 (2)	C19—O21	1.3300 (16)
C1—C10	1.4210 (19)	O21—C22	1.4628 (16)
C1—H1A	0.9500	C22—C23	1.496 (2)
C2—C3	1.418 (2)	C22—H22A	0.9900
C2—H2A	0.9500	C22—H22B	0.9900
C3—C4	1.420 (2)	C23—H23A	0.9800
C3—C8	1.420 (2)	C23—H23B	0.9800
C4—C5	1.373 (2)	C23—H23C	0.9800
C4—H4A	0.9500	C24—C25	1.4749 (18)
C5—C6	1.404 (2)	C25—N27	1.2893 (17)
C5—H5A	0.9500	C25—C26	1.4991 (18)
C6—C7	1.366 (2)	C26—H26A	0.9800
C6—H6A	0.9500	C26—H26B	0.9800
C7—C8	1.415 (2)	C26—H26C	0.9800
C7—H7A	0.9500	N27—N28	1.3700 (15)
C8—C9	1.4182 (19)	N28—C29	1.3587 (17)
C9—C10	1.3682 (19)	N28—H28A	0.8800
C9—H9A	0.9500	C29—C34	1.4130 (19)
C10—C12	1.4998 (18)	C29—C30	1.4160 (18)
C12—O13	1.4214 (16)	C30—C31	1.3898 (18)
C12—H12A	0.9900	C30—N35	1.4524 (17)
C12—H12B	0.9900	C31—C32	1.3699 (19)
O13—C14	1.4181 (16)	C31—H31A	0.9500
C14—C15	1.4930 (19)	C32—C33	1.3945 (19)
C14—H14A	0.9900	C32—N38	1.4596 (17)
C14—H14B	0.9900	C33—C34	1.3684 (18)
C15—O16	1.3554 (16)	C33—H33A	0.9500
C15—C24	1.3575 (19)	C34—H34A	0.9500
O16—N17	1.3950 (15)	N35—O36	1.2227 (14)
N17—C18	1.3120 (17)	N35—O37	1.2443 (14)
C18—C24	1.4291 (18)	N38—O39	1.2282 (15)
C18—C19	1.4876 (19)	N38—O40	1.2284 (15)
C19—O20	1.2047 (16)		
			
C2—C1—C10	120.75 (13)	C19—O21—C22	114.54 (11)
C2—C1—H1A	119.6	O21—C22—C23	107.90 (12)
C10—C1—H1A	119.6	O21—C22—H22A	110.1
C1—C2—C3	120.89 (13)	C23—C22—H22A	110.1
C1—C2—H2A	119.6	O21—C22—H22B	110.1
C3—C2—H2A	119.6	C23—C22—H22B	110.1
C2—C3—C4	122.80 (13)	H22A—C22—H22B	108.4
C2—C3—C8	118.70 (13)	C22—C23—H23A	109.5
C4—C3—C8	118.50 (13)	C22—C23—H23B	109.5
C5—C4—C3	120.92 (14)	H23A—C23—H23B	109.5
C5—C4—H4A	119.5	C22—C23—H23C	109.5
C3—C4—H4A	119.5	H23A—C23—H23C	109.5
C4—C5—C6	120.20 (14)	H23B—C23—H23C	109.5
C4—C5—H5A	119.9	C15—C24—C18	103.34 (11)
C6—C5—H5A	119.9	C15—C24—C25	125.47 (12)
C7—C6—C5	120.32 (14)	C18—C24—C25	131.16 (12)
C7—C6—H6A	119.8	N27—C25—C24	113.88 (11)
C5—C6—H6A	119.8	N27—C25—C26	124.68 (12)
C6—C7—C8	121.02 (14)	C24—C25—C26	121.28 (11)
C6—C7—H7A	119.5	C25—C26—H26A	109.5
C8—C7—H7A	119.5	C25—C26—H26B	109.5
C7—C8—C9	121.98 (13)	H26A—C26—H26B	109.5
C7—C8—C3	119.02 (13)	C25—C26—H26C	109.5
C9—C8—C3	118.99 (13)	H26A—C26—H26C	109.5
C10—C9—C8	121.36 (12)	H26B—C26—H26C	109.5
C10—C9—H9A	119.3	C25—N27—N28	115.76 (11)
C8—C9—H9A	119.3	C29—N28—N27	119.06 (11)
C9—C10—C1	119.31 (12)	C29—N28—H28A	120.5
C9—C10—C12	122.27 (12)	N27—N28—H28A	120.5
C1—C10—C12	118.41 (12)	N28—C29—C34	120.13 (11)
O13—C12—C10	109.06 (10)	N28—C29—C30	122.70 (12)
O13—C12—H12A	109.9	C34—C29—C30	117.16 (12)
C10—C12—H12A	109.9	C31—C30—C29	121.66 (12)
O13—C12—H12B	109.9	C31—C30—N35	116.43 (11)
C10—C12—H12B	109.9	C29—C30—N35	121.90 (11)
H12A—C12—H12B	108.3	C32—C31—C30	118.64 (12)
C14—O13—C12	114.09 (10)	C32—C31—H31A	120.7
O13—C14—C15	113.35 (11)	C30—C31—H31A	120.7
O13—C14—H14A	108.9	C31—C32—C33	121.72 (12)
C15—C14—H14A	108.9	C31—C32—N38	119.48 (12)
O13—C14—H14B	108.9	C33—C32—N38	118.76 (12)
C15—C14—H14B	108.9	C34—C33—C32	119.63 (12)
H14A—C14—H14B	107.7	C34—C33—H33A	120.2
O16—C15—C24	109.91 (11)	C32—C33—H33A	120.2
O16—C15—C14	118.15 (11)	C33—C34—C29	121.19 (12)
C24—C15—C14	131.86 (12)	C33—C34—H34A	119.4
C15—O16—N17	109.21 (10)	C29—C34—H34A	119.4
C18—N17—O16	105.26 (10)	O36—N35—O37	122.13 (11)
N17—C18—C24	112.27 (12)	O36—N35—C30	118.81 (11)
N17—C18—C19	120.58 (12)	O37—N35—C30	119.06 (10)
C24—C18—C19	127.11 (12)	O39—N38—O40	123.59 (11)
O20—C19—O21	124.76 (12)	O39—N38—C32	118.01 (11)
O20—C19—C18	122.48 (12)	O40—N38—C32	118.39 (11)
O21—C19—C18	112.76 (11)		

### Hydrogen-bond geometry (Å, °)

**Table:**

*D*—H···*A*	*D*—H	H···*A*	*D*···*A*	*D*—H···*A*
N28—H28A···O37	0.88	1.96	2.6028 (14)	128
